# Isolation, Characterization and Transcriptome Analysis of a Cytokinin Receptor Mutant *Osckt1* in Rice

**DOI:** 10.3389/fpls.2017.00088

**Published:** 2017-01-31

**Authors:** Wona Ding, Huishan Tong, Wenjuan Zheng, Jing Ye, Zhichong Pan, Botao Zhang, Shihua Zhu

**Affiliations:** ^1^College of Science and Technology, Ningbo UniversityNingbo, China; ^2^School of Marine Sciences, Ningbo UniversityNingbo, China; ^3^Cixi Institute of Biomedical Engineering, Ningbo Institute of Materials Technology and Engineering, Chinese Academy of SciencesNingbo, China

**Keywords:** cytokinin, histidine kinase, transcriptome, rice, Oryza sativa

## Abstract

Cytokinins play important roles in regulating plant development, including shoot and root meristems, leaf longevity, and grain yield. However, the *in planta* functions of rice cytokinin receptors have not been genetically characterized yet. Here we isolated a rice mutant, *Osckt1*, with enhanced tolerance to cytokinin treatment. Further analysis showed that *Osckt1* was insensitive to aromatic cytokinins but responded normally to isoprenoid and phenylurea-type cytokinins. Map-based cloning revealed that the mutation occurred in a putative cytokinin receptor gene, histidine kinase 6 (*OsHK6*). *OsCKT1* was found to be expressed in various tissues throughout the plant and the protein was located in the endoplasmic reticulum. In addition, whole-genome gene expression profiling analysis showed that *OsCKT1* was involved in cytokinin regulation of a number of biological processes, including secondary metabolism, sucrose and starch metabolism, chlorophyll synthesis, and photosynthesis. Our results demonstrate that *OsCKT1* plays important roles in cytokinin perception and control of root development in rice.

## Introduction

Cytokinin, a class of adenine derivatives, regulates many processes in plants, such as tissue and organ development and response to environmental stimuli (Mok and Mok, 2001; Werner and Schmulling, 2009). Cytokinin signaling is transducted by a two-component system (TCS) through a His-to-Asp phosphorelay (Heyl and Schmulling, 2003; Kakimoto, 2003). TCSs were originally identified in bacteria, and in the basal form they involve a receptor kinase that autophosphorylates on a conserved His residue in response to an environmental stimulus and the phosphate is then transferred to a conserved Asp residue of a response regulator (RR), which subsequently modulates its downstream signaling in the pathway (Stock et al., 2000; Gao and Stock, 2009). In plants the TCS consists of a multistep phosphorelay, i.e., His to Asp to His to Asp (Hutchison and Kieber, 2002; Sheen, 2002; Kakimoto, 2003; Ferreira and Kieber, 2005). Cytokinin is first perceived by hybrid histidine kinase (HK) receptors, mainly localized in endoplasmic reticulum (ER), and results in autophosphorylation (Anantharaman and Aravind, 2001; Caesar et al., 2011; Lomin et al., 2011; Wulfetange et al., 2011). After an intramolecular phosphotransfer, the phosphorylation signal is transmitted to His-containing phosphotransfer proteins (HPs), and then they translocate to the nucleus (Punwani et al., 2010). In the nucleus, type-B RRs are activated by HPs through phosphorylation and they subsequently initiate the transcription of their target genes, including type-A RRs, a class of negative regulators of cytokinin signaling (Hwang and Sheen, 2001; Deruere and Kieber, 2002; To et al., 2004).

The system is extensively studied mainly in *Arabidopsis*. Three HKs, AHK2, AHK3, and AHK4, function redundantly as cytokinin receptors (Inoue et al., 2001; Ueguchi et al., 2001; Higuchi et al., 2004). The *ahk2 ahk3* mutant showed fewer leaf cells, reduced chlorophyll content, a strongly enhanced root system and increased branching, and the *ahk2 ahk3 ahk4* mutant showed severe shoot and root defect and very low fertility, with a reduction in meristem size and activity, seeds of the *ahk2 ahk3 ahk4* mutant were more than twice as large as wild-type (WT; Higuchi et al., 2004; Riefler et al., 2006). A number of *Arabidopsis* RRs (ARRs) have also been characterized. Over-expression study of all type-A RRs found that they were regulated by both the cytokinin and proteasome pathways and executed distinctive functions in plant growth and development (Ren et al., 2009). ARR15 and ARR16 were shown to play distinct roles in roots (Kiba et al., 2002, 2003). Over-expression of *ARR22* in *Arabidopsis* resulted in dwarf phenotypes and poorly developed root systems (Kiba et al., 2004). RR2 was shown to control leaf longevity through cytokinin-mediated phosphorylation (Kim et al., 2006). Three type-B RRs, ARR1, ARR10, and ARR12, were found to be key players in cytokinin regulation of root protoxylem differentiation, lateral root formation, chlorophyll levels, and cytokinin primary response (Mason et al., 2005; Yokoyama et al., 2007). *arr1 arr10 arr12* plants showed very severe growth defect which was highly analogous to the *ahk2 ahk3 ahk4* triple mutant (Ishida et al., 2008).

The conserved cytokinin TCS also exist in rice (Ito and Kurata, 2006; Du et al., 2007; Schaller et al., 2007). The rice genome is predicted to have four HKs, two HPs, 13 type-B RRs and 10 type-A RRs. However, the signaling pathway is relatively less characterized in rice compared to *Arabidopsis*. Only a few components were genetically characterized in detail. The two HPs in rice were found to be positive regulators of the cytokinin signaling pathway and played different roles in salt and drought tolerance (Sun et al., 2014). Over-expression of *OsRR6* resulted in dwarf phenotypes with poorly developed root systems and panicles (Hirose et al., 2007). Recently, OsHK3 was reported to play a role in the regulation of ABA-induced antioxidant defense and in the feedback regulation of H_2_O_2_ production in ABA signaling when transiently expressed in protoplasts (Wen et al., 2015). Another study also proposed that OsHK3 was similar to the osmosenser AtHK1 in *Arabidopsis* based on protein structure analysis (Kushwaha et al., 2014). OsHK6 was found to be a cytokinin receptor with preferential affinity for isopentenyladenine (iP; Choi et al., 2012). However, there is still no genetic characterization of rice cytokinin receptors reported yet.

Here we provided the first piece of genetic evidence to show the *in planta* function of a cytokinin receptor in rice using a loss-of-function mutant. We also conducted whole-genome expression profiling study to analyze its role in cytokinin regulation of rice root development.

## Materials and Methods

### Plant Materials and Growth Conditions

The *Osckt1* mutant was isolated from an ethyl methanesulfonate mutagenized (EMS)-mutagenized rice mutant library (*Oryza sativa* L. *Japonica* cv Xiushui63) in culture solution prepared as described with 0.2 μM 6-Benzylaminopurine (BA; Yoshida et al., 1976). After being germinated in water for 2 days in the dark, phenotypic characterization of the WT and mutant was performed in a growth chamber at 30/22°C (day/night) and 60–70% humidity with a photoperiod of 12 h. More than 30 plants of each genotype were used for each treatment.

### Mapping and Cloning of *OsCKT1*

A mapping population of 225 F_2_ plants was generated from crosses between the homozygous *Osckt1* mutant and Kasalath (*Oryza sativa* L. *Indica*). F_2_ plants exhibiting short roots and abolished lateral roots were selected for mapping of the mutant locus of *OsCKT1. OsCKT1* was mapped using SSR markers. The candidate gene between markers flanking both sides of the mutant locus was amplified by PCR from both the WT and *Osckt1* plants for sanger sequencing analysis. The genomic DNA and mRNA regions of the *OsCKT1* gene were amplified and sequenced using the forward and reverse primers 5′-GGGGAAGAAGGAGGAGGAGTAGATT and 5′-CCACTAGCCAGACCATCATCATACC.

### Construction of Vectors and Plant Transformation

The coding region of *OsCKT1* mRNA was isolated by PCR amplification with the primers *OsCKT1F Kpn*I (5′-AAAGGTACCGGGGAAGAAGGAGGAGGAGTA) and *OsCKT1R Sal*I (5′-AAAGTCGACCCACTAGCCAGACCATCATCA). The PCR product was ligated into the pUCM-T vector (Takara) and sequenced. Then the fragment was excised from the pUCM-T vector by *Kpn*I and *Sal*I digestion and ligated into the corresponding site of pCAMBIA1301(35S). A 2071 bp promoter of *OsCKT1* was obtained by PCR using primers: 5′-AAAGTCGACTTATTGCCCAAAATGCCCCTC (containing a *Sal*I recognition site) and 5′-AAAGGTACCATCCCCCCTCCCTCTCAGAAAT (containing a *Kpn*I recognition site). The resulting DNA fragment was inserted into the vector pCAMBIA1300NH-GUS via *Sal*I/*Kpn*I sites to create a transcriptional fusion of the *OsCKT1* promoter and the β-glucuronidase (GUS) coding sequence, *OsCKT1p::GUS*. The above constructs were used for *Agrobacterium tumefaciens-*mediated rice transformation of WT or mutant materials as described (Chen et al., 2003).

### Histochemical GUS Staining Analysis

Histochemical GUS analysis was performed as previously described (Ding et al., 2015). Transgenic plant samples and freehand cross-sections of the stem base were incubated with GUS staining solution (100 mmol l^-1^ NaH_2_PO_4_ buffer pH 7.0, 0.5% Triton X-100, 0.5 mg ml^-1^ X-Gluc, and 20% methanol) overnight at 37°C. Then, tissues were mounted on slides and photographed (Leica MZ95, Nussloch, Germany).

### Subcellular Localization of OsCKT1

The full-length coding sequence of *OsCKT1* with the eliminated stop codon was inserted in-frame before the coding sequence of a soluble modified green fluorescent protein (smGFP4). The OsCKT1-GFP fusion coding sequence was subcloned into the binary vector 35S-pCAMBIA1301. The resulting construct was sequenced to verify in-frame fusion and used for transient transformation of onion epidermis using a gene gun (Bio-Rad, Hercules, CA, USA). PHF1-RFP located to the ER was co-transformed (Chen et al., 2011). The GFP and RFP were visualized using a LSM 510 laser-scanning microscope (Zeiss, Jena, Germany).

### RNA extraction, cDNA Library Preparation, and Digital Expression Profiling

Total RNA was extracted from roots of 8-day old WT and *Osckt1* under 0.2 μM BA treatment using the RNeasy Plant Mini Kit (Qiagen, USA). Two biological replicates from each genotype were used for RNA-sequencing. RNA was quantified using the Nanodrop-2000 (ThermoFisher, USA) and RNA quality was then examined using a 2100 Bioanalyzer (Agilent Technologies, USA). High-quality RNA samples for library construction were selected based on 260/280 nm ratio and RNA integrity number (RIN) above 2.0 and 8.0, respectively. Sequencing libraries were prepared using the NEBNext Ultra RNA Library Prep Kit for Illumina (NEB, USA) according to the manufacturer’s instructions. Poly-A-containing mRNA from the total RNA was isolated, purified, and fragmented. After the first and second strand cDNA synthesis and adaptor ligation, double-stranded cDNAs were then purified for end repair, dA tailing, adaptor ligation, and enrichment. Libraries were subjected to 75 cycles of single-end sequencing with the Illumina Nextseq 500 system (Illumina, USA) according to the manufacturer’s instructions. The raw sequencing data have been uploaded to the SRA (Sequence Read Archive^1^) database (accession number: SRP091783).

### Differentially Expressed Gene Analysis

Raw reads of fastq format were firstly processed through in-house perl scripts. In this step, clean data were obtained by removing reads containing adapter, reads containing ploy-N and low quality reads (the rate of reads which quality value < = 30 is more than 50%). The retained high-quality clean reads, i.e., clean reads, were then analyzed by the TopHat-Cufflinks pipeline (Trapnell et al., 2012). Briefly, cleans reads were mapped to the rice genome (MSU version 7^2^) using TopHat. Cufflinks was then used for transcriptome assembly and assessment of the FPKM value and expression difference between two genotypes. Genes differentially expressed by at least twofold between the two genotypes with a FDR adjusted *P*-value < 0.05 were assigned as differentially expressed genes (DEGs). For gene ontology (GO) enrichment analysis, the singular enrichment analysis (SEA) tool in agriGO (Du et al., 2010) was applied with default parameters and a threshold FDR adjusted *P*-value < 0.05. The function categorization of DEGs was conducted by MapMan (Thimm et al., 2004). The Kyoto Encyclopedia of Genes and Genomes (KEGG) pathway enrichment analysis was conducted by PlantGSEA (Yi et al., 2013).

## Results

### Isolation and Genetic Analysis of *Osckt1*

**FIGURE 1:**
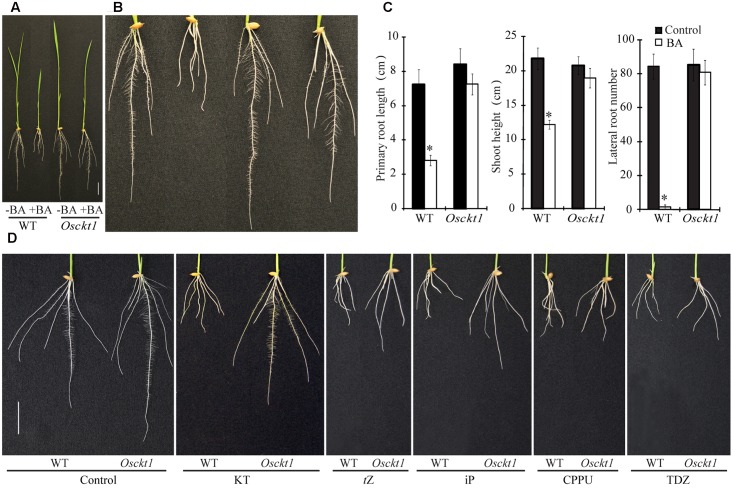
**Phenotypic characterization of the wild-type (WT) and *Osckt1*.** **(A)** Growth of 8-day-old seedlings of the WT and *Osckt1* without or with 0.2 μM 6-Benzylaminopurine (BA) treatment. **(B)** Enlarged view of roots of the WT and *Osckt1* from **(A)**. **(C)** Effect of BA treatment on lateral root number, shoot height, and primary root length of the WT and *Osckt1*. Significant differences were determined using Student’s *t*-test (^∗^*P* < 0.05). **(D)** Root growth of 8-day-old WT and *Osckt1* under treatments of 0.2 μM KT, 0.05 μM *t*Z, 0.1 μM iP, 0.02 μM CPPU, and 0.02 μM TDZ. Bars = 2 cm.

In order to identify molecular components of the cytokinin signaling pathway in rice, a forward genetic screening was conducted using an ethylmethane sulfonate (EMS)-mutagenized rice mutant library (*Oryza sativa* L. *Japonica* cv. Xiushui63). One mutant with decreased sensitivity to cytokinin was isolated and designated c*ytokinin tolerant 1* (*Osckt1*). Under control condition, the growth of *Osckt1* was similar to the WT (**Figure 1A**). WT plants responded to 6-benzyladenine (BA) with significantly inhibited root and shoot growth, and especially almost no lateral root at 8 days after germination (**Figures 1A–B**). By contrast, such response almost disappeared in *Osckt1* (**Figures 1A–C**). When treated with BA, root and shoot growth of *Osckt1* was only slightly but not significantly reduced, and the initiation and growth of lateral roots was comparable to control condition.

The response of *Osckt1* to other cytokinins was also analyzed, including kinetin (KT), *trans*-Zeatin (*t*Z), iP, *N*-(2-chloro-4-pyridyl)-*N*′-phenylurea (CPPU) and thidiazuron (TDZ). Among them, *Osckt1* was found to be insensitive to KT only compared with the WT (**Figure 1D**).

### Cloning of *OsCKT1*

**FIGURE 2:**
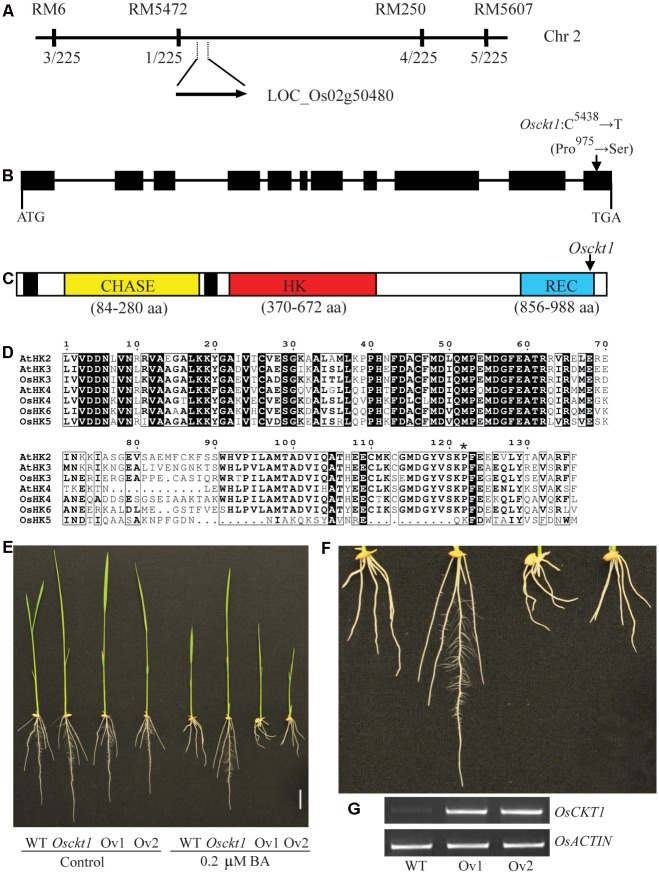
**Map-based cloning of *OsCKT1*.** **(A)** *OsCKT1* was mapped to a 2137 kb region on chromosome 2. The rates of recombinants in the F_2_ population are listed below the molecular markers. **(B)** Gene structure of *OsCKT1*. Black boxes represent exons, and lines indicate introns and the 5′ and 3′ untranslated regions. The point mutation in the last exon is indicated. **(C)** Secondary structure analysis of OsCKT1 using the CDD database (https://www.ncbi.nlm.nih.gov/cdd). Black boxes represent transmembrane domains. The point mutation results in a substitution of Proline by Serine in the REC domain. **(D)** Protein sequence alignment of the REC domain of HKs from *Arabidopsis* and rice. Identical and highly similar amino acids are highlighted in black and framed in black by ESPript, respectively. The mutated residue in *Osckt1* was indicated by asterisk. **(E**–**G)** Complementation analysis of the *Osckt1* mutant. Two independent lines of over-expression transgenic plants (Ov1 and Ov2) in the *Osckt1* mutant background were displayed **(E)** and the enlarged view of roots under BA treatment was shown **(F)**. RT-PCR analysis **(G)** of *OsCKT1* in roots of Ov1 and Ov2. Bar = 2 cm.

A F_2_ population was developed by crossing the *Osckt1* mutant with the WT Kasalath (*Indica*). The F_1_ seedlings showed similar sensitivity to BA as the WT and their F_2_ progenies displayed segregation of WT and *Osckt1* phenotypes at a ratio of 3:1 (148:52, χ^2^ = 0.35 < χ^2^_0.05_,_1_ = 3.84, *P* > 0.05), indicating that the short root phenotype in *Osckt1* is controlled by a single recessive nuclear gene. The *OsCKT1* locus was first mapped to chromosome 2 between SSR markers RM5 and RM5607 using 30 F_2_ mutant plants (**Figure 2A**). Two new markers were used for fine mapping using 225 F_2_ mutant plants. The *OsCKT1* gene was further mapped to a 2137 kb region between RM5472 and RM250 (**Figure 2A**).

The genome sequence of rice between these markers was searched for genes that could code for proteins involved in cytokinin signal transduction. Among them there was one hypothetical gene, LOC_Os02g50480, possibly encoding a cytokinin receptor, HK6. It was considered as a highly possible candidate for *OsCKT1* and Sanger sequencing analysis for this gene in both the WT and *Osckt1* was conducted. One single point mutation within the gene in *Osckt1* was identified. The *OsCKT1* gene is 5509 bp in length, and contains 11 exons and 10 introns, respectively (**Figure 2B**). The point mutation (C^5438^ to T) occurring at the last exon of the gene resulted in an amino acid substitution (Pro^975^ to Ser). The protein coding region of *OsCKT1* is 2994 bp and encodes a 997 amino acid protein. The protein structure is consistent with the annotation generated by the CDD database (Conserved Domain Database^3^), with two transmembrane domains, a CHASE domain, a HK domain, and a REC domain (**Figure 2C**).

The CHASE domain is an extracellular cytokinin sensor and the HK domain is a dimerization and phosphoacceptor domain. Besides a phosphorylatable Asp residue for receiving the phosphoryl group from the HK domain, the REC domain is responsible for formation of homodimers or heterodimers with HPs in the process of phosphorelay and a highly conserved Pro residue within the domain was thought to be involved in formation of the hydrophobic dimerization surface (Müller-Dieckmann et al., 1999; Solà et al., 1999). Protein sequence alignment analysis of the REC domain of three *Arabidopsis* and four rice cytokinin receptors showed that the Pro residue was highly conserved (**Figure 2D**). The mutation of Pro^975^ in *Osckt1* corresponding to the conserved critical residue might explain its dramatic effect on the function of OsCKT1. It was also worth noting that the REC domain of OsHK5 only contained the first half conserved region and the other half was significantly different from others, suggesting its putatively diverged function (**Figure 2D**).

### Complementation Test of *Osckt1*

Complementation analysis of the *Osckt1* mutant was conducted using *Agrobacterium tumefaciens*-mediated transformation. The 2994 bp protein coding region of *OsCKT1* was cloned into the pCAMBIA 1301 vector driven by the 35S promoter and used for transformation of *Osckt1*. More than twenty independent transgenic lines were obtained. The sensitivity of these transformants to BA was restored (**Figures 2E–F**). Insertion and expression of the transgene in two representative lines were confirmed by RT-PCR (**Figure 2G**).

### Expression Pattern and Subcellular Localization Analysis of OsCKT1

**FIGURE 3:**
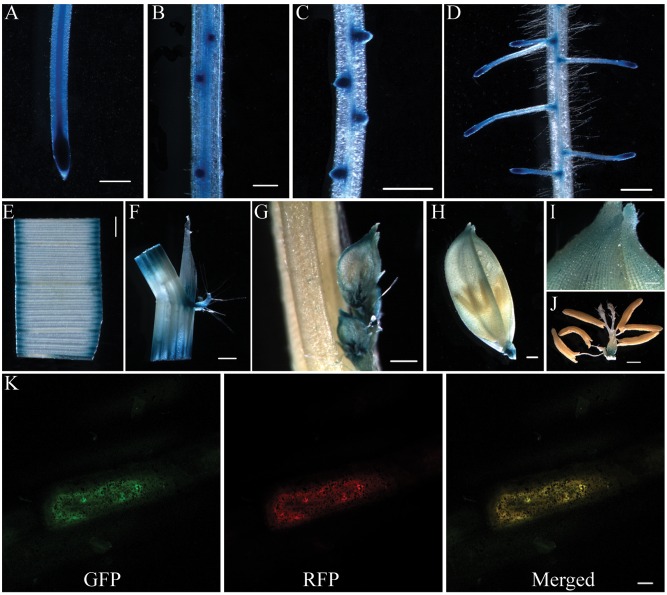
**Expression pattern of *OsCKT1* and subcellular localization of OsCKT1.** **(A–J)** Promoter-β-glucuronidase (GUS) fusion studies reveal the expression of *OsCKT1* in various tissues, root tip **(A)**, lateral root primordium **(B)**, emerging lateral roots **(C)**, lateral root tips **(D)**, leaf **(E)**, stem, ligule, and auricle **(F)**, young spikelet **(G)**, glume **(H,I)**, and flower **(J)**. **(K)** OsCKT1 targets green fluorescent protein (GFP) to ER in transiently transformed onion epidermal cells. The PHF1-RFP is used as the endoplasmic reticulum (ER) marker. **(A**,**C–F**,**H**,**J**) Bars=0.5 mm; **(B**,**G**,**I)** Bars=0.2 mm; **(K)** Bar = 20 μm.

To determine the expression pattern of *OsCKT1*, a 2071 bp promoter sequence before the protein coding region of the *OsCKT1* gene was fused to the GUS reporter gene. This chimeric gene cassette was use to transform WT plants via the *Agrobacterium tumefaciens*-mediated transformation method. Histochemical staining for GUS activity in T_2_ plants showed that *OsCKT1* was ubiquitously expressed in plant organs, including the root, leaf, stem, ligule, auricle, young spikelet, glume, and flower (**Figures 3A–J**). Strong expression was observed in root tips, lateral root primordia, emerging lateral roots, and lateral root tips (**Figures 3A–D**).

To examine the subcellular localization of OsCKT1, a chimeric fusion gene of coding region of *OsCKT1* and the green fluorescent protein (GFP) under the control of the 35S promoter was constructed and delivered into onion epidermal cells for transient expression. Fluorescence analysis showed that the fusion protein co-localized with a co-transformed ER marker (**Figure 3K**), indicating that OsCKT1 located in the ER.

### Whole-Genome Expression Analysis of *Osckt1* under Cytokinin Treatment

**FIGURE 4:**
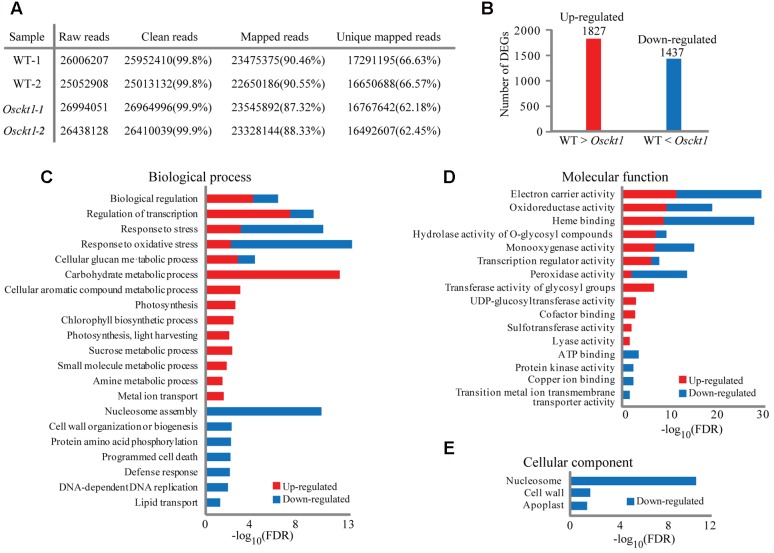
**Analysis and gene ontology (GO) enrichment of differentially expressed genes (DEGs) between the WT and *Osckt1* under BA treatment by RNA-seq.** **(A)** General information of sequencing reads and mapping. **(B)** The number of up- and down-regulated DEGs between the WT and *Osckt1*. **(C–E)** GO term enrichment analysis of up- and down-regulated DEGs in Biological process **(C)**, Molecular function **(D)**, and Cellular component **(E)**.

To further gain insight into the *in planta* function of *Osckt1* in cytokinin signaling, digital gene expression profiling analysis of WT and *Osckt1* under BA treatment was conducted using RNA-seq. Two replicates of each genotype were used, yielding four libraries in total. Each of these libraries generated more than 25 million 75-bp single-end reads after quality control and about 62∼66% of them were uniquely mapped onto the rice reference genome (**Figure 4A**). A total of 3264 DEGs were identified with a cut-off of log_2_(fold change) > = 1 and FDR adjusted *P*-value < 0.05. Among them, 1827 DEGs showed higher expression in the WT than *Osckt1* and were termed as up-regulated genes, while 1437 DEGs showed lower expression in the WT than *Osckt1* and were termed as down-regulated genes (**Figure 4B**; **Supplementary Table S1**).

To classify the function of DEGs, GO enrichment analysis was performed separately for up- and down-regulated DEGs using agriGO (Du et al., 2010). Within the biological process category, up-regulated DEGs were largely associated with regulation, response to stress, carbohydrate metabolism, and photosynthesis, while down-regulated DEGs were largely associated with regulation, response to stress, nucleosome assembly, cell wall, protein phosphorylation, and cell death (**Figure 4C**). Congruent with this, within the molecular function term both up- and down-regulated DEGs were associated with electron carrier, oxidoreductase, and heme binding (**Figure 4D**). Furthermore, up-regulated DEGs were specifically associated with transferase activity, while down-regulated DEGs with ATP binding and kinase activity. No up-regulated DEGs were enriched within the cellular component GO term, however, down-regulated DEGs showed association with nucleosome, cell wall, and apoplast (**Figure 4E**).

**FIGURE 5:**
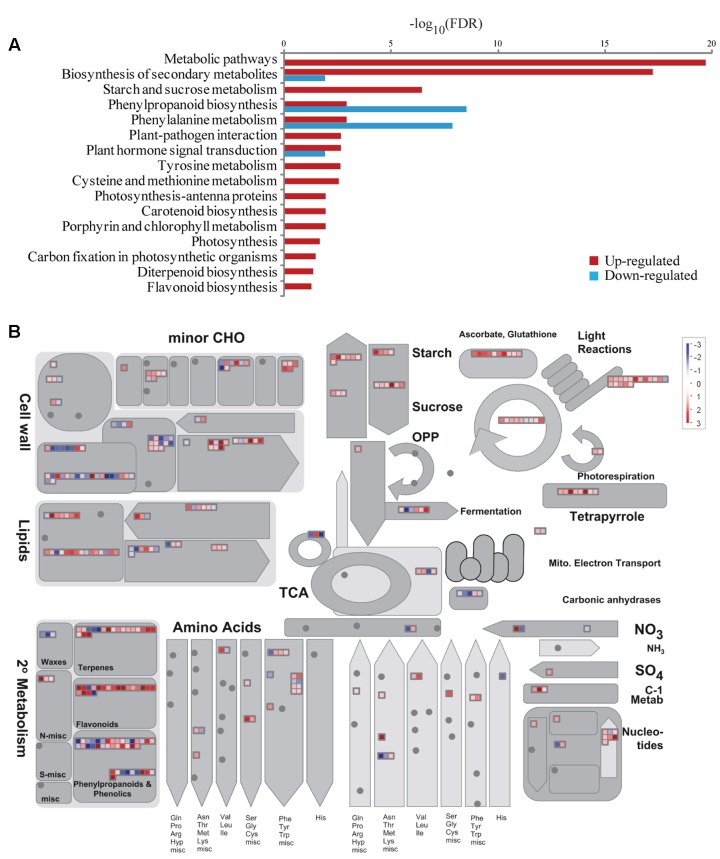
**Pathway enrichment and MapMan overview of DEGs between the WT and *Osckt1* under BA treatment.** **(A)** Kyoto Encyclopedia of Genes and Genomes (KEGG) pathway enrichment of up- and down-regulated DEGs. **(B)** Overview of all DEGs involved in metabolic processes by MapMan.

**FIGURE 6:**
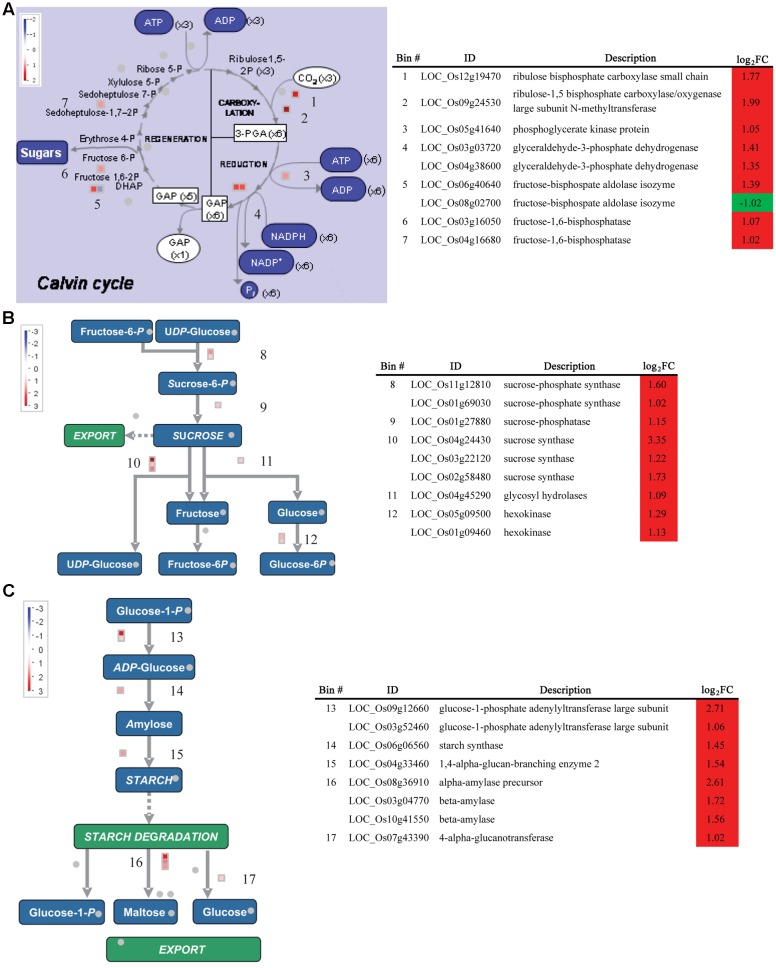
**Expression of DEGs between the WT and *Osckt1* under BA treatment associated with the Calvin cycle and Starch and Sucrose metabolism.** **(A–C)** DEGs associated with the Calvin cycle **(A)**, sucrose **(B)**, and starch metabolism **(C)** are shown, with a table showing gene names, putative functions, and fold change. The values in red and blue indicate log_2_-transformed fold increase and decrease in expression, respectively.

Kyoto Encyclopedia of Genes and Genomes pathway enrichment analysis was also conducted to identify significantly affected metabolic or signal transduction pathways among DEGs. Sixteen and four pathways were significantly enriched for up- and down-regulated DEGs, respectively (FDR < 0.05; **Figure 5A**). Those pathways for up-regulated DEGs mostly correlated to amino acid metabolism, secondary metabolite synthesis, hormone signaling, and photosynthesis, while down-regulated DEGs showed enrichment in phenylpropanoid biosynthesis, phenylalanine metabolism, and hormone signaling. Analysis of all DEGs using MapMan software^4^ further showed that DEGs involved in photosynthesis and sucrose and starch metabolism were mostly up-regulated (**Figure 5B**). These genes were examined in more detail. In total 22 DEGs were mapped to most steps in the calvin cycle, which is responsible for carbon fixation and sucrose and starch metabolism pathways, among which only one gene was down-regulated, suggesting up-regulation of these pathways under BA treatment (**Figures 6A–C**).

## Discussion

In the present study, a rice mutant *Osckt1* was isolated from a rice EMS-mutagenized population and the mutation caused loss of function of OsHK6, a cytokinin receptor. OsHK6 was proposed to be the rice ortholog of CRE1 in *Arabidopsis* (Du et al., 2007). Similar to its *Arabidopsis* counterpart, the mutant did not show any obvious growth defect under normal condition (Higuchi et al., 2004). It was previously reported that OsHK6 showed preferential affinity for iP (Choi et al., 2012). However, the *in planta* function of OsHK6 has not been clearly characterized. The loss-of-function mutant of *OsHK6* exhibited insensitivity to BA and KT with normal root and shoot growth, especially normal lateral root initiation and elongation when treated with them, suggesting that OsHK6 was the major if not the only receptor for BA and KT, two aromatic cytokinins, in rice. We further conducted rice whole-genome digital gene expression profiling analysis to compare the gene expression between WT and *Osckt1* under BA treatment. GO, MapMan, and KEGG analysis showed that DEGs were significantly associated with a number of biological processes, including metabolism, hormone signal transduction, stress response, photosynthesis, etc.

Genes involved in amino acid metabolism and secondary metabolism were significantly responsive to cytokinin (**Figures 5A,B**). They mainly belong to two groups. One is the synthesis of phenolic secondary metabolites, including tyrosine and phenylalanine metabolism, biosynthesis of phenylpropanoid and flavonoids. It’s clearly that phenylalanine metabolism and biosynthesis of phenylpropanoid both significantly enriched in the up- and down-regulated gene list by BA, indicating the complex regulation of the process. The other is biosynthesis of terpenoid, including diterpenoid and carotenoid. These results suggest that cytokinin may induce the synthesis of these compounds, which is consistent with previous reports (Deikman and Ulrich, 1995; Bhargava et al., 2013; Akagi et al., 2014).

The homeostasis of cytokinin is tightly regulated to coordinate the signaling pathways in plants (Sakakibara, 2006; Hirose et al., 2008). Adenosine phosphate isopentenyltransferase (IPT) catalyzes the fist and rate-limiting step of CK biosynthesis (Sakamoto et al., 2006). Then the products are hydroxylated by a cytochrome P450 monooxygenase CYP72A1 (Sakakibara, 2006). LOGs, a class of cytokinin-activating enzyme, work in the final step of bioactive cytokinin synthesis and produce free-base form of cytokinin, such as iP and tZ (Kurakawa et al., 2007). Cytokinin is then perceived by HKs, which initiate intracellular phosphotransfer to subsequent RRs (Higuchi et al., 2004). Besides, OsCKXs are cytokinin oxidase/dehydrogenases, and cytokinin glucosyltransferases catalyze the inactivation of cytokinin by *O*-glucosylation, which are responsible for removal of over-accumulated active cytokinin by degradation or conjugation, respectively (Ashikari et al., 2005; Sakakibara, 2006; Kudo et al., 2010, 2012). In our study, the expression of one *IPT* gene (LOC_Os03g59570), two *CYP72A1* genes (LOC_Os09g23820, LOC_Os08g33300), and two *LOG* genes (*OsLOGL3*, *OsLOGL10*) were significantly down-regulated by BA treatment, suggesting the overall repression of cytokinin biosynthesis (**Table 1**). Concurrent with this, three cytokinin dehydrogenase genes (*OsCKX2*, *OsCKX4*, *OsCKX5*) and 13 putative cytokinin glucosyltransferase genes were significantly up-regulated by BA treatment (**Table 1**). Moreover, two genes (*OsPUP3*, *OsPUP4*) encoding purine permeases (PUPs)were induced and repressed, respectively. Some members of the PUP family were involved in cytokinin transport (Bürkle et al., 2003; Qi and Xiong, 2013). Several type-A *RRs* (*OsRR4*, *OsRR6*, *OsRR9*, *OsRR10*) were induced by BA treatment (**Table 1**). Type-A RRs were mainly found to be negative regulators of cytokinin signaling pathway and over-expressing *RR6* in rice resulted in dwarf phenotypes with poorly developed root systems (Hirose et al., 2007). The results suggest that the perception of BA in the WT may initiate the feedback signaling pathway to repress BA biosynthesis and signaling and promote BA inactivation, which help to counteract the overaccumulation of BA and maintain internal homeostasis, while in *Osckt1* the inability to respond to BA treatment made these unnecessary.

**Table 1:** Selected differentially expressed genes (DEGs) associated with hormones.

Category	ID	Gene	Description	log_2_(FC)
Cytokinin				
Biosynthesis	LOC_Os05g47840	*IPT7*	IPP transferase	2.14
	LOC_Os03g59570	*IPT4*	IPP transferase	-1.11
	LOC_Os09g23820	*CYP72A1*	Cytochrome P450 72A1	-2.78
	LOC_Os08g33300	*CYP72A1*	Cytochrome P450 72A1	-3.35
	LOC_Os05g51390	*LOGL8*	Uncharacterized protein PA4923	1.11
	LOC_Os03g01880	*LOGL3*	Possible lysine decarboxylase domain containing protein	-1.56
	LOC_Os10g33900	*LOGL10*	Possible lysine decarboxylase domain containing protein	-3.76
	LOC_Os01g10110	*CKX2*	Cytokinin dehydrogenase precursor	5.26
	LOC_Os01g71310	*CKX4*	Cytokinin dehydrogenase precursor	1.12
	LOC_Os01g56810	*CKX5*	Cytokinin dehydrogenase precursor	3.13
Conjugation	LOC_Os04g46980	*cisZOG1*	*Cis*-zeatin *O*-glucosyltransferase	1.72
	LOC_Os04g46970		Glucosyltransferase	1.52
	LOC_Os02g36830		Cytokinin-*O*-glucosyltransferase 2	4.14
	LOC_Os04g37820		Cytokinin-*O*-glucosyltransferase 2	2.36
	LOC_Os04g25440		Cytokinin-*O*-glucosyltransferase 2	1.50
	LOC_Os02g28900		Cytokinin-*O*-glucosyltransferase 2	1.04
	LOC_Os04g37820		Cytokinin-*O*-glucosyltransferase 2	2.36
	LOC_Os04g25440		Cytokinin-*O*-glucosyltransferase 2	1.50
	LOC_Os02g11130		Cytokinin-*O*-glucosyltransferase 3	1.13
	LOC_Os04g44250		Cytokinin-*O*-glucosyltransferase 3	-1.98
	LOC_Os07g13810		Cytokinin-*N*-glucosyltransferase 1	1.18
	LOC_Os07g13810		Cytokinin-*N*-glucosyltransferase 1	1.18
	LOC_Os01g59100		Cytokinin-*N*-glucosyltransferase 1	1.57
Transport	LOC_Os09g29239	*PUP3*	Purine permease	1.40
	LOC_Os01g48800	*PUP4*	Purine permease	-2.49
Signaling	LOC_Os02g58350	*RR3*	OsRR3 type-A response regulator	-1.10
	LOC_Os01g72330	*RR4*	OsRR4 type-A response regulator	1.15
	LOC_Os04g57720	*RR6*	OsRR6 type-A response regulator	1.50
	LOC_Os11g04720	*RR9*	OsRR9 type-A response regulator	1.25
	LOC_Os12g04500	*RR10*	OsRR10 type-A response regulator	1.26
Auxin				
Biosynthesis	LOC_Os01g16714	*YUCCA10*	Flavin monooxygenase	-1.12
	LOC_Os01g51060		IAA-amino acid hydrolase	1.22
	LOC_Os01g55940	*GH3.2*	OsGH3.2 – Probable indole-3-acetic acid-amido synthetase	-1.10
Transport	LOC_Os11g04190	*PIN1c*	Auxin efflux carrier component	-1.80
	LOC_Os09g38210	*PILS7b*	Auxin efflux carrier component	3.98
	LOC_Os09g38130	*PILS7a*	Auxin efflux carrier component	1.58
	LOC_Os01g50080	*MDR9*	MDR-like ABC transporter	2.24
	LOC_Os03g14080	*AUX3*	Transmembrane amino acid transporter protein	-1.61
	LOC_Os10g05690	*AUX4*	Transmembrane amino acid transporter protein	-1.72
Transduction	LOC_Os01g56240	*SAUR2*	OsSAUR2 – Auxin-responsive SAUR gene family member	1.80
	LOC_Os02g05050	*SAUR4*	OsSAUR4 – Auxin-responsive SAUR gene family member	1.29
	LOC_Os02g07110	*SAUR6*	OsSAUR6 – Auxin-responsive SAUR gene family member	3.10
	LOC_Os02g20320	*SAUR7*	OsSAUR7 – Auxin-responsive SAUR gene family member	-1.85
	LOC_Os04g51890	*SAUR20*	OsSAUR20 – Auxin-responsive SAUR gene family member	-1.71
	LOC_Os04g52670	*SAUR21*	OsSAUR21 – Auxin-responsive SAUR gene family member	1.54
	LOC_Os06g48850	*SAUR27*	OsSAUR27 – Auxin-responsive SAUR gene family member	-1.45
	LOC_Os06g50040	*SAUR29*	OsSAUR29 – Auxin-responsive SAUR gene family member	1.03
	LOC_Os09g37410	*SAUR46*	OsSAUR46 – Auxin-responsive SAUR gene family member	1.60
	LOC_Os02g04810	*ARF5*	auxin response factor 5	1.33
	LOC_Os02g49160	*IAA8*	OsIAA8 – Auxin-responsive Aux/IAA gene family member	1.27
	LOC_Os03g58350	*IAA14*	OsIAA14 – Auxin-responsive Aux/IAA gene family member	1.35
	LOC_Os06g07040	*IAA20*	OsIAA20 – Auxin-responsive Aux/IAA gene family member	1.96
	LOC_Os06g39590	*IAA23*	OsIAA23 – Auxin-responsive Aux/IAA gene family member	1.13
Ethylene				
Biosynthesis	LOC_Os11g08380		1-aminocyclopropane-1-carboxylate oxidase	-2.76
	LOC_Os05g05670		1-aminocyclopropane-1-carboxylate oxidase	2.50
	LOC_Os06g14390		1-aminocyclopropane-1-carboxylate oxidase homolog 4	1.27
	LOC_Os08g30100		1-aminocyclopropane-1-carboxylate oxidase homolog 1	1.03
	LOC_Os01g35230		1-aminocyclopropane-1-carboxylate oxidase homolog 1	-1.89
	LOC_Os09g27750		1-aminocyclopropane-1-carboxylate oxidase 1	3.64
	LOC_Os02g53180		1-aminocyclopropane-1-carboxylate oxidase protein	2.55
	LOC_Os05g05680		1-aminocyclopropane-1-carboxylate oxidase	1.58
	LOC_Os03g51740	*ACS1*	Aminotransferase, classes I and II, domain containing protein	2.39


Other hormones, such as auxin and ethylene, are known to closely interact with cytokinin to regulate plant development (Moubayidin et al., 2009; Schaller et al., 2015). Cytokinin suppresses auxin signaling by altering the expression of auxin efflux carrier PIN genes to control auxin transport, redistribution and downstream signaling (Staswick et al., 2005; Hwang et al., 2012; Marhavy et al., 2014). Cytokinin also induces the biosynthesis of ethylene and thus inhibits root elongation through ethylene signaling, where auxin biosynthesis, transport, signaling, and response are required (Chae et al., 2003; Stepanova et al., 2007; Ruzicka et al., 2009). In our study there are a number of auxin-related genes differentially regulated by BA treatment, including genes for auxin synthesis (YUCCA10, GH3.2, LOC_Os01g51060), transport (PIN1c, PILS7a, PILS7b, MDR9, AUX3, AUX4), and several SAUR gene family members and AUX/IAA genes for signal transduction (**Table 1**). *OsGH3.2* encodes an IAA-amino synthetase which conjugates excess IAA to amino acids to suppress the action of IAA (Fu et al., 2011). Previous study in *Arabidopsis* showed that cytokinins repress *PIN1* expression in lateral root founder cells and abolish the formation of an auxin gradient required for lateral root primordium patterning (Laplaze et al., 2007; Marhavy et al., 2011). Similarly, *OsPIN1c* was found to be specifically expressed in the root meristem, stele, and lateral root primordia (Wang et al., 2009). OsIAA23 was previously reported to be critical for root development, and its constitutive activation resulted in the absence of lateral and crown root primordia (Ni et al., 2011). The repression of *OsPINc* and the induction of *OsIAA23* in the WT by BA treatment might play key roles in the observed defects of root development. Moreover, one ACC synthase and six ACC oxidase genes involved in ethylene synthesis were up-regulated in the WT by BA treatment (**Table 1**), which might result in the activation of ethylene signaling pathways and subsequently lead to inhibition of root elongation through auxin.

Chlorophyll biosynthesis and chloroplast biogenesis are positively regulated by cytokinin signaling and negatively regulated by auxin signaling in *Arabidopsis* roots (Kobayashi et al., 2012). Treatment of rice leaves by BA induce the expression of genes in the chlorophyll cycle and PSII-related genes, resulting in delay of senescence and the stability of photosynthetic pigment complexes (Talla et al., 2016). Consistent with this, GO enrichment and KEGG enrichment analysis in our study both showed the over-representation of genes involved in photosynthesis, chlorophyll synthesis in the up-regulated gene list (**Figures 4C** and **5A**).

Furthermore, a number of genes involved in the Calvin cycle and starch and sucrose metabolism were up-regulated (**Figures 5B** and **6A–C**). For the Calvin cycle, those up-regulated genes encode rubisco-related components, phosphoglycerate kinase, glyceraldehyde-3-phosphate dehydrogenase, fructose-bisphosphate aldolase, fructose-1,6-bisphosphatase, and sedoheptulose-bisphosphatase, respectively. Two genes encoding the sucrose–phosphate synthase, the key enzyme in sucrose synthesis, and one gene encoding sucrose phosphatase were highly induced by BA treatment. In addition, gene encoding sucrose synthase, glycosyl hydrolases, and hexokinase showed significant increase in expression under BA treatment. Hexokinases are sugar sensors and lead to glucoses entering the glycolytic pathway (Jang et al., 1997). Besides, genes encoding enzymes involved in both starch synthesis and degradation, namely ADP glucose pyrophosphorylase, starch synthase, starch-branching enzyme and amylase, were significantly induced by BA treatment. Overall, these data suggest an up-regulation of sucrose and starch biosynthesis and degradation by BA treatment.

In brief, we characterized the *in planta* function of a cytokinin receptor, OsHK6, using a loss-of-function mutant. Our results showed that the mutation caused tolerance of root development to BA and kinetin, suggesting that they are specifically perceived by OsHK6 *in vivo*. We further conducted rice whole-genome digital gene expression profiling to elucidate the underlying molecular mechanism and identified enriched functional groups involved in chlorophyll synthesis and carbon fixation, starch and sucrose metabolism, secondary metabolite synthesis, hormone signal transduction, etc. These results may improve our understanding of the functions of cytokinin signaling pathways in rice root development.

## Author Contributions

WD, BZ, and SZ conceived and designed the study. WD, HT, WZ, JY, ZP, and BZ performed the experiments. WD and BZ analyzed data and wrote the manuscript. All authors read and approved the manuscript.

## Conflict of Interest Statement

The authors declare that the research was conducted in the absence of any commercial or financial relationships that could be construed as a potential conflict of interest.

## Footnotes

1 https://www.ncbi.nlm.nih.gov/sra

2 http://rice.plantbiology.msu.edu/pub/data/Eukaryotic_Projects/o_sativa/annotation_dbs/pseudomolecules/version_7.0/

3 https://www.ncbi.nlm.nih.gov/cdd

4 http://mapman.gabipd.org
